# Associations Between Childhood Sexual Abuse, Intimate Partner Violence Trauma Exposure, Mental Health, and Social Gender Affirmation Among Black Transgender Women

**DOI:** 10.1089/heq.2022.0161

**Published:** 2023-11-27

**Authors:** JaNelle M. Ricks, Jessica Horan

**Affiliations:** ^1^ College of Public Health Division of Health Behavior and Health Promotion, The Ohio State University, Columbus, Ohio, USA.; ^2^ College of Public Health Division of Epidemiology, The Ohio State University, Columbus, Ohio, USA.

**Keywords:** gender identity, transgender, mental health, prevention, HIV/AIDS

## Abstract

**Introduction:**

The purpose of this study was to examine associations between social gender affirmation and mental health outcomes. The resulting relationships were explored within the context of childhood sexual abuse (CSA) and intimate partner violence (IPV) trauma history.

**Materials and Methods:**

A community sample of 138 Black trans women completed structured interviews assessing gender affirmation, mental health, and trauma history. Bivariate associations between gender affirmation scale and mental health measures were assessed using Pearson coefficients. Multiple linear regression models were used to generate adjusted estimates of association.

**Results:**

Childhood sexual victimization and IPV were common. Depressive symptoms, anxiety, quality of life, and body image satisfaction were significantly associated with social gender affirmation. Recent IPV dampened this association to not significant. Gender affirmation and anxiety/panic and quality-of-life outcomes did not retain significance in models adjusted for lifetime IPV. CSA did not weaken the association.

**Conclusion:**

Incorporating trauma-informed and gender-affirmative frameworks into medical care and evidence-based interventions is a crucial structural step toward protection of the mental health of Black trans women.

## Introduction

Trans women, those assigned male sex at birth, but who identify as a woman, face a unique set of health challenges compared with cisgender (non-transgender) peers. Stigma, transphobia, trauma, and systemic inequity marginalize these women, and such exposures increase the risk of engagement in high-risk behaviors (e.g., survival sex, condomless anal sex), and contribute to mental health morbidities.^1,2^ Disproportionately higher rates of poor mental health outcomes, including elevated risk of psychological distress, attempted suicide, substance abuse, and weight and shape control behaviors, are commonly reported within this population.^3,4^

For Black trans women, who live at the intersection of marginalized race and gender identities historically battered by discriminatory social and economic conditions and abusive acts and attitudes, the risk of poor mental health is compounded. The health disparities that exist between Black trans women and their counterparts who do not endorse multiple minoritized identities are not simply caused by race or gender, but by the legacies of oppression manifested as colonialism, racism, patriarchy, transphobia, and sociopolitical barriers to optimal health that lead to exposures and behaviors known to contribute to disease and disability.^5,6^ Intersectionality, a sociological framework derived from Black feminist theory and praxis,^7^ provides important framing to understand how interlocking axes of structural power and privilege (e.g., racism, sexism, classism) present as unique differences in interpersonal and individual experiences for people occupying intersectional positions (i.e., Black trans women).^8^

Intersectionality posits that social identities and related inequalities depend upon and construct one another.^9^ A particular combination of marginalized social categories creates a subjective experience that is unique from what is created by the combination of any fewer of those statuses, including distinctive experiences of discrimination.

Although there is a paucity of population health research illuminating the mental health experiences of Black trans women,^10^ existing evidence demonstrates that dual experiences of transphobia and racism directly shape the needs and access of these women.^11^ Intersectional racial and gendered discrimination is associated with an estimated two-to-seven times the odds of morbidities such as experiencing post-traumatic stress disorder,^12^ depressive symptoms,^13^ suicide attempts,^6^ and body dissatisfaction and weight and shape control behaviors.^14,15^ For those able to scale the substantial barriers to access of services, gender and race dually heighten experiences of self-internalized transmisogyny, which then limits effectiveness of mental health services.^16,17^

The pathway from structural oppression and discrimination to mental health problems among sexual and gender minority people is elucidated by the minority stress theory. The impact of cumulative minority stress—the “excess stress to which individuals from stigmatized social categories are exposed as a result of their social, often a minority, position”^6^—has been correlated with the high prevalence of poor mental health outcomes among transgender people.^18^ According to this model, general environmental factors (e.g., education level) and one's minority identity limit access to external resources and internal coping mechanisms.

Building on this framework to more acutely account for the unique stressors experienced by transgender people, the gender minority stress framework posits three stressor types: distal stressors (e.g., gender-based victimization), negative expectations (e.g., identity concealment), and internalized transphobia (i.e., internalization of negative societal attitudes about one's identity or transgender people), which have been correlated with stress-related mental health problems.^18,19^

Intuitively, the oppression faced by those who hold a racial minority identity carries with it a unique set of stressors, also shown to increase risk of poor health outcomes. Intersectional gender minority stress work has shown this effect to be amplified for those who endorse both gender and racial minoritized identities because of the overlapping forms of oppression with which they are confronted.^5,20,21^

Despite this evidence, however, the literature does not consistently show a prevalence of mental health disorders for gender minorities of color.^5^ This suggests that other factors, such as social safety, may also have a role.^22^ Social safety refers to core human needs (i.e., reliable social connection, inclusion, and protection) that are harmed by stigma. Building on the minority stress model linking social stigmatization to health outcomes, this theory posits that the chronic threat-vigilance fostered by insufficient safety has negative long-term effects on cognitive, emotional, and immunological functioning, even when exposure to minority stress is low.^22^

Regarding physical, emotional, and sexual trauma, trans women of color face increased vulnerability to intimate partner violence (IPV) and sexual abuse because of harmful societal stereotypes that specifically position them as hypersexual and stigmatize their relationships with cisgender men.^23,24^ They also experience childhood sexual abuse (CSA) at a rate that far exceeds data on women in the general U.S. population.^25,26^ The detrimental mental health effects of CSA for Black trans women survivors are elevated.^27^

Gender affirmation (GA) (an interpersonal, interactive process whereby a person receives social recognition and validation of gender identity and expression^1^) is a framework through which social and personal factors safeguarding transgender health can be understood. It is multidimensional, comprising four core constructs: psychological (i.e., internal sense of self-actualization), medical (e.g., hormones, surgery, other body modification), legal (e.g., legal name change), and social.^28^ Social GA can include living full or part time in one's identified gender, dressing in ways that align with one's gender identity, and disclosing one's gender/transgender experience to others. Social GA is often the first or only form of GA that trans women engage in.^29^

Several studies have linked social GA experiences to positive mental health in diverse transgender populations.^26,30,31^ This satisfaction may provide increased protection against the impact of stigma, discrimination, or other forms of victimization, leading to improved health outcomes. Attending to the constructs of social GA may be key to promotion of mental health of trans women,^32^ particularly Black trans women as those with higher social GA report elevated acceptance of self and life and lower experiences of depressive symptoms, anxiety, and suicidal ideation.^33^ The increase in social acceptance and social support associated with GA alleviates the distress associated with gender identity and body incongruence, ultimately improving the mental health of trans women.^33^

This study's purpose was to (1) examine associations between social GA and mental health outcomes and (2) explore the resulting relationships between social GA and mental health, in the context CSA and IPV history.

## Materials and Methods

### Participants and procedures

This analysis uses secondary data from a community-based sexual health study conducted among trans women. A community sample of Black trans women living in Atlanta, GA, and Chicago, IL, were recruited using multiple community-based strategies: word of mouth by advocates at venues serving trans women and advertisement through formal and informal communication channels in LGBTQ advocacy groups and service organizations.

Eligibility criteria: (1) identify as Black; (2) 18 to 65 years of age; (3) assigned male sex at birth; (4) self-identify as transgender (male-to-female) or female; and (5) report anal sex with a cisgender male in past six months. We recognize that “male-to-female” is dated and potentially stigmatizing language and that “transfeminine” is more appropriate. However, because this term was used in the instrument, it was appropriate to leave the references to that language in the manuscript when describing the survey. Procedures to be taken in response to distress or discomfort (e.g., skipping the question, directing to support services) were discussed. After providing written consent, participants completed a structured interview during which a staff member trained in qualitative methods orally administered the survey in a private room. Women received $30 incentives for participation. Study protocols were approved by the Georgia State University Institutional Review Board. Data were collected between April 2014 and June 2017.

### Measures

#### Sociodemographic characteristics

Sociodemographic characteristics assessed included age, race, self-reported HIV status, highest level of education completed, current employment status, income, history of involvement with criminal justice system, recent housing history, and food insecurity.

#### Social GA

Congruence between appearance and gender identity and comfort with disclosing gender/transgender experience to others was measured. The 10-item scale (Cronbach's alpha=0.72) was previously validated with a sample of Black trans women.^34^ Example item: (1) I am happy with the way my appearance expresses my gender identity. Five-point Likert scale was used (1=strongly agree and 5=strongly disagree) for response options. Five scale items were reverse coded. Higher scores represent a higher degree of affirmation.

### Mental Health Outcomes

#### Psychological distress

The 10-item Perceived Stress Scale^35^ (Cronbach's alpha=0.77) has been used in previous work with trans individuals.^36–38^ Example item: “in the last month, how often have you felt that you were unable to control the important things in your life.” Five-point Likert scale was used (1=never and 5=very often) for response options. Dimension score range: 5–25. Higher scores represented greater stress experiences.

#### Depressive symptoms

The five-item depressive symptom subscale from the Brief Symptom Inventory^39^ (Cronbach's alpha=0.83) assessed symptoms in the past 7 days. Example item: (1) “feeling lonely.” Five-point Likert scale was used (1=not at all and 5=extremely) for response options. Dimension score range: 5–25. Higher scores indicated more depressive symptomology.

#### Anxiety and panic

The six-item anxiety subscale from the Brief Symptom Inventory^39^ (Cronbach's alpha=0.91) assessed symptoms experienced in the past 7 days. Example item: “suddenly scared for no reason.” Five-point Likert scale was used (1=not at all and 5=extremely) for response options. Dimension score range: 6–30. Higher scored indicated more significant symptoms.

#### Quality of life

The five-item Satisfaction with Life Scale^40^ (Cronbach's alpha=0.86) was used. Example item: “in most ways I feel my life is close to ideal.” Five-point Likert scale was used (1=strongly agree and 5=strongly disagree) for response options. Dimension score range: 5–25. Larger numbers indicated greater life satisfaction.

#### Body image satisfaction

A previously validated seven-item subscale^41^ (Cronbach's alpha=0.83) was used. Example item: “how happy are you with your face.” Five-point Likert scale was used (1=very unhappy and 5=very happy) for response options. Dimension score range: 7–35. Higher scores represented higher degree of satisfaction.

### Trauma history variables

#### Childhood sexual abuse

Assessed with the following item: “as a child (less than 16 years old), were you ever sexually abused.” Response options: yes, no, I do not remember, dichotomized into “yes” and “no,” with “I do not remember” responses (*n*=2) folded into the “no” category.

#### Twelve-month IPV

The 5-item subscale of the National Intimate Partner and Sexual Violence Survey^42^ was used. Example item: “in the past 12 months, has a romantic or sexual partner physically abused you.” Response options: yes or no.

#### Lifetime IPV

The 5-item subscale of the National Intimate Partner and Sexual Violence Survey^42^ was used. Example item: “in your lifetime, has someone ever made you perform oral sex.” Response options: yes or no.

### Data analysis

Bivariate associations between the social GA scale and each of the five mental health measures were assessed using Pearson coefficients. Statistically significant associations were subsequently entered into multiple linear regression models to generate adjusted estimates of association. Models were adjusted for age, HIV status, study site, housing history, incarceration history, socioeconomic status (education, employment, income, and food insecurity^43^), and trauma history, all selected based on demonstrated association with study outcomes.^44^ Due to the size of the study sample, significance was defined by a *p-*value of <0.10.

## Results

### Sample characteristics

The sample of 138 Black trans women ranged in age from 18 to 65 years (mean=30.8; SD=10.0). More than half (58.7%) reported public or private health insurance coverage. Almost half (44.5%) reported homelessness in the past 12 months. Half (46.4%) reported positive HIV status. Education beyond high school had been attained by 40.6%. The mean score on the social GA scale was 7.18 (SD=2.88), with a range of 4 to 18. Please see Table 1 for additional results.

**Table 1.** Characteristics of Sample of Black Transgender Women (*N*=138)

	***N*** (%)	***M*** (SD)
Age: range (18–65)		30.8 (10.0)
Living with HIV	64 (46.4)	
Highest level of education		
Some high school	22 (15.9)	
High school diploma/GED	60 (43.5)	
At least some college	52 (37.7)	
Post-graduate degree	4 (3.6)	
Current employment status		
Full-time employment	26 (18.8)	
Part-time employment	17 (12.3)	
Full-time student	5 (3.6)	
Disabled for work	19 (13.8)	
Unemployed	70 (50.7)	
Current health insurance coverage	81 (58.7)	
Geographic location		
Atlanta	77 (55.8)	
Chicago	61 (44.2)	
Experienced homelessness in past 12 months	61 (44.2)	
Lifetime incarceration history	75 (54.3)	
Trauma history		
Childhood sexual abuse	56 (40.6)	
IPV experienced during last 12 months		
Forced to perform oral sex	16 (11.6)	
Forced receptive anal sex	19 (13.8)	
Forced insertive anal sex	13 (9.4)	
Physical abuse	20 (14.5)	
Emotional abuse	35 (25.4)	
Lifetime IPV experienced		
Forced to perform oral sex	56 (40.6)	
Forced receptive anal sex	58 (42.0)	
Forced insertive anal sex	25 (18.1)	
Physical abuse	62 (44.9)	
Emotional abuse	87 (63.0)	
Mental health outcomes		
Psychological stress: range (10–46)		30.30 (6.87)
Depression: range (5–25)		10.63 (4.81)
Anxiety and panic: range (6–30)		10.63 (5.34)
Quality of life: range (5–24)		12.96 (4.71)
Body image: range (7–35)		26.89 (6.19)

IPV, intimate partner violence.

Trauma experiences were common among this sample of women (Table 1). Almost half (40.6%) reported CSA victimization. Lifetime physical and emotional abuse perpetrated by a romantic or sexual partner were the most commonly experienced trauma (44.9% and 63.0%). Lifetime experiences with forced oral sex and receptive anal sex were also high (40.6%, 42.0). Table 1 displays scale means of mental health outcomes. Mean body image satisfaction scores indicated a higher degree of body satisfaction in this sample. Participants also reported high levels of psychological stress.

### Bivariate associations

Analyses determined that four of the five mental health outcomes tested were significantly associated with the social GA measure (Table 2). The coefficients for associations between social GA and these outcomes were significant, with directions suggesting protective effects (i.e., positive mental health outcomes were associated with higher ratings of social GA).

**Table 2.** Bivariate Associations Between Social Gender Affirmation and Mental Health Outcomes for a Sample of Black Transgender Women (*N*=138)

Outcome measure	Gender affirmation ***r (p)***
Psychological stress	−0.06 (0.45)
Depression	**−0.16 (0.04)**
Anxiety and panic	**−0.20 (0.01)**
Quality of life	**0.18 (0.02)**
Body image	**0.25 (.002)**

Bold indicates *p*-value <0.05.

### Multivariate associations

Table 3 displays the adjusted Beta estimates for associations between social and the four mental health outcome measures. After adjusting for age, HIV status, study site, housing history, incarceration history, and socioeconomic status, the relationship between social GA and mental health outcomes maintained a significant association. In the presence of these six sociodemographic covariates and 12-month IPV experience, the association was weakened to nonsignificant. The sociodemographic covariates plus CSA did not weaken the significance in association between social GA and any of the mental health outcomes. The relationship between social GA and the anxiety/panic and quality-of-life outcomes did not retain significance in models adjusted for the sociodemographic covariates and lifetime trauma. Significance was retained for the relationships between social GA and depressive symptoms and social GA and body image satisfaction in models also adjusted for these covariates.

**Table 3.** Beta Estimates for Models of Social Gender Affirmation, Mental Health, and Trauma Experiences for a Sample of Black Transgender Women (*N*=138)

	No trauma variables		Childhood sexual abuse ***B*** (***p***)		12-month IPV ***B*** (***p***)		Lifetime IPV ***B*** (***p***)	
	***B*** (***p***)	adj. ***B*** (***p***)	***B*** (***p***)	adj. ***B*** (***p***)	***B*** (***p***)	adj. ***B*** (***p***)	***B*** (***p***)	adj. ***B*** (***p***)
Depression	0.16 (0.04)	0.17 (.07)	0.15 (0.06)	0.18 (0.06)	0.13 (0.39)	0.07 (0.66)	0.18 (0.03)	0.17 (0.09)
Anxiety and panic	0.20 (0.01)	0.16 (0.10)	0.11 (0.17)	0.16 (0.10)	0.29 (0.04)	0.05 (0.76)	0.24 (0.004)	0.12 (0.20)
Quality of life	**0.18 (0.02)**	**0.16 (0.10)**	0.01 (0.95)	**0.17 (0.09)**	0.06 (0.69)	0.04 (0.84)	**0.15 (0.07)**	0.14 (0.17)
Body image	**0.25 (0.002)**	**−0.29 (0.002)**	0.09 (0.28)	**−0.30 (0.002)**	0.13 (0.37)	0.004 (0.98)	**0.16 (0.05)**	**−0.32 (<0.001)**

Models have been adjusted for age, HIV status, study site, housing history, incarceration history, socioeconomic status, and trauma history. Bold indicates *p*-value ≤0.10.

Exploration of the relative contribution of trauma history to the regression models tested provided varying results. In both models in which the addition of lifetime trauma covariates diminished the significance in association between social GA and mental health outcomes (i.e., anxiety/panic, quality of life), trauma made the largest contribution, followed by unemployment status. In models in which the addition of past 12-month IPV covariates diminished the significance in association between social GA and mental health outcomes (i.e., depressive symptoms, anxiety/panic, quality of life, body image satisfaction), the set of covariates providing the largest contributions had no observable pattern, but included combinations of trauma, lower education attainment, unemployment status, homelessness, justice system involvement, and positive HIV status.

## Discussion

A primary aim of this study was to examine the relationship between social GA and mental health outcomes among Black trans women. The association was strong for all but one of the mental health outcomes tested, suggesting that a social GA framework may support efforts to improve mental health of these women. This aligns with prior studies that have demonstrated a positive association between social GA and optimal mental health of trans women.^26,31^

A particularly strong and perhaps intuitive relationship between social GA and body image satisfaction was observed, emphasizing a link between a woman's comfort with the external presentation of a congruent gender identity and her satisfaction with the internalized mental representation of her body. Trans women are likely to be affected by the narrative of gendered beauty ideals broadcast in popular culture and public discourse. The stakes for conforming to societal standards of femininity are high not only because of the gender-related social stressors^45,46^ but also because “passing” as non-transgender can be necessary for managing daily safety and survival.^1,47^ Cis male partners often use coercive control, violence, and perpetrate homicide against trans women to conceal socially stigmatized romantic and sexual relationships from others.^24^

Elevated economic and social adversity (e.g., economic instability, employment discrimination, biological family rejection) experienced by trans women also increase dependency on abusive partners for economic or social support.^48^ Furthermore, Black trans women routinely experience victim blaming (often explicitly related to race, clothing, or gender expression), while seeking help post-IPV exposure.^49^ Research on racism, racial stereotyping, and rape victim shows that blaming occurs more frequently among victims of inter-racial rape compared to victims of intraracial rapes.^50^ This is particularly salient for trans women of color who are targeted and killed at a disproportionate rate in the ongoing epidemic of violence against the transgender community.^51^

In addition, gender socialization and gender transition for these women require negotiating both Western femininity ideals (thinness, whiteness, and youthfulness) and the competing ideals of femininity rooted within their own racial identity and community norms.^35^ Inclusion of a social GA framework in the provision of mental health care and interventions to prevent negative outcomes and health disparities for Black trans women may be increasingly effective, but use should consider both dominant and culturally specific determinants of health.

The second aim of this study was to explore the relationship between social GA and mental health, in the context of trauma history. Exposure to CSA and IPV was commonly experienced across the life course. This is consistent with theory and research explaining the adverse mental health impacts of chronic and intersectional minority stress that Black trans women endure.^6,7,52–54^ The relationship observed between social GA and mental health was weakened by discrete types of trauma exposure. Across all mental health outcomes, treating recent IPV (i.e., experienced in the last 12 months) as a covariate, diminished the relationship between social GA and mental health to an association that was not significant. This perhaps indicates a temporal effect of IPV exposure on mental health, washing out any positive effect of social GA.

Due to the cross-sectional nature of data collection, however, we were unable to establish a temporal or causal association. There are significant associations between IPV and mental health outcomes such as depressive symptoms and anxiety, but findings reported in the literature have been inconsistent.^55^ Measuring lifetime exposure to IPV may be insufficient to properly explore the complexity of these relationships because estimates of exposure are heterogeneous. Recent IPV exposure, however, has consistently been shown to have a bidirectional relationship with depressive symptoms.^55^ Additional research is needed to better understand these relationships for Black trans women, as well as any protective effect of GA.

Among women reporting ever experiencing trauma, this nonsignificant pattern repeats for social GA and the anxiety/panic and quality-of-life outcomes, but not depressive symptoms and body image satisfaction. This perhaps indicates that of the mental health outcomes explored, depressive symptoms and body image satisfaction operationalize mental health quality most robustly, and that the effect of social GA on mental health has less benefit for women with an accumulation of trauma.

Alternatively, anxiety/panic symptoms and quality of life may more closely approximate the threat-vigilance and avoidance associated with post-traumatic stress disorder. This could have been more salient than the negative experience of depressive symptoms. A similar pattern was observed among women reporting a history of CSA. Controlling for CSA did not weaken the significance in association between social GA and any of the mental health outcomes. The other three GA dimensions (i.e., medical, legal, psychological) were not explored in this study, but may interact with trauma history and mental health differently than social GA.

Although a 2020 study of social and medical GA among trans-masculine, trans-feminine, and gender nonbinary adults in the United States demonstrated that both social GA and medical GA were inversely associated with depressive and anxiety and stress symptoms, results were not presented by race and gender.^49^ For Black trans women, it is probable that because they experience more barriers to health care access, including medical GA procedures and care, compared with White trans women, overcoming said barriers and engaging in medical affirmation may be more clinically meaningful for mental health than achieving high social affirmation.^56^ Future study of multiple dimensions of GA among Black trans women is needed to illuminate this further.

### Limitations

Participants were recruited for a sexual health study, which excluded those who had not been sexually involved with a cisgender man in the preceding 6 months. In addition, a majority of women were recruited through community-based organizations providing support and services to trans women, which also introduces selection bias.

Potential impact of this bias' effect may be gleaned from studies of sexual minority health, which demonstrated that community venue samples tend to underrepresent individuals with lower income and those who are unemployed.^57^ This omission is concerning as it perpetuates longstanding disparities in health status by socioeconomic status among sexual and gender minorities. Also, this representation issue threatens the accurate and complete identification of target samples, limiting generalizability. The cross-sectional study design limits the ability to determine temporal or causal relationships. Longitudinal conceptualization of participants' gender transition process was not captured; women who have been transitioning over a longer period may have had more time to experience benefits, including higher social GA and better mental health outcomes, compared with women in earlier stages of transition.^58^

Assessing CSA using one item may not have been sufficient to fully determine exposure. Finally, the positionality of the researcher innately impacts their understanding and interpretation of findings.^59^ The authors of this article include sexually diverse (queer and straight), and racially diverse (Black, White, and Latina) cisgender and queer graduate students and public health experts in sexual and gender minority health. Throughout this work, we attempted to reflexively recognize our positioning outside the experiences of women who participated. We acknowledge that despite this, our positions may have limited nuanced understanding and each of us may have blind spots that impacted the clarity and accuracy of our interpretation.

### Health equity implications

This study has at least three implications for health equity in practice. First, social GA is an important framework to use in addressing the health-related needs of Black trans women who face layered forms of oppression that increase their risk for disproportionate psychiatric morbidity. In particular, empirical studies have demonstrated a link between social GA and improvements in mental health,^60,61^ and thus its inclusion should be considered in approaches targeting and supporting these women. Kaplan et al. demonstrated this application by using a gender-affirmative model and community connectedness and social cohesion constructs in their mental and sexual health intervention adapted for trans women. In doing so, they were able to observe fewer poor mental and sexual health outcomes (e.g., depression scores, sexually transmitted infection incidence).^62^

Second, addressing structural and social stigma that create inequities for Black trans women remains critical as these exposures will continue to undermine mental health prevention, treatment, and care efforts if left unattended. Trans women of color are underrepresented in samples of clinical research, indicating unequal access to care. A recent political assault against transgender social and structural protections further threatens this access. During the first 4 months of 2023, 492 anti-transgender bills across 47 states have been introduced. This is a 2489% increase since 2015.^63^ Studies with sexual minority populations demonstrate the substantive benefit from progressive changes in distal structures such as policy on the health of stigmatized groups.^64,65^ These structures cannot be ignored as they heavily influence availability of options given to Black trans women who are marginalized to positions of sociostructural disadvantage.^66^

Finally, the high prevalence of trauma among this population calls for an integration of trauma-informed approaches into prevention and care and research settings.^67^ A trauma-informed approach includes the following: (1) acknowledgment of diffuse impacts of trauma and various routes to recovery; (2) recognition that specific signs and symptoms may be associated with experiences of trauma; (3) responses that are comprehensive and integrative; and (4) efforts made to prevent re-traumatization.^68,69^ To be most effective, it is imperative that this approach “emphasizes safety; trustworthiness and transparency; peer support; collaboration and mutuality; and empowerment, voice, and choice; and addresses cultural, historical, and gender issues.”^68^ As an example, providers can undertake antistigma work to educate themselves about the lived experiences of these women and approach them with respect and sensitivity.^16^ Reisner et al.'s informed consent model of trans health care serves salient modeling of this best practice.^70^

By incorporating a trauma-informed framework into medical care and evidence-based interventions, providers and researchers may help increase resilience against negative experiences, thereby expanding the capacity of women to achieve positive mental health outcomes.

## Conclusions

For Black trans women, the association between social GA and mental health is both strong and positive. This relationship, however, is weakened by trauma exposure. Considering both the elevated risk of trauma exposure and the marked mental health risks posed by discrimination, our findings stand in league with evidence emphasizing protective effects of social GA. In addition to gender-affirmative frameworks, incorporating trauma-informed approaches into medical care, evidence-based research, and public health interventions is another crucial step toward structural protection of the mental health of Black trans women.

## Abbreviations Used

CSA: childhood sexual abuse
GA: gender affirmation
IPV: intimate partner violence
